# Characterization of pathogenic microorganisms in diabetic foot infections and development of a risk prediction model

**DOI:** 10.1038/s41598-025-07092-5

**Published:** 2025-07-02

**Authors:** Shufang Yan, Xuemeng Zhu, Tong Yu, Dandan Li, Su Li, Lei Xue

**Affiliations:** 1 Department of Endocrinology, Henan University Huaihe Hospital, Kaifeng, 475500 Henan China; 2 Department of Pathology, Henan University Huaihe Hospital, Kaifeng, Henan China

**Keywords:** Diabetic foot infection, Pathogenic microorganism, Risk prediction model, Diseases, Endocrinology, Medical research

## Abstract

This study aimed to investigate the distribution of pathogenic microorganisms in diabetic foot infections (DFIs) and develop a nomogram to predict DFIs. It included 136 diabetic foot (DF) patients hospitalized at Henan University Huaihe Hospital from November 2020 to November 2024, with 86 (63.23%) having confirmed infections. Infections were predominantly caused by *Gram-positive cocci* (54.65%) and *Gram-negative bacilli* (43.02%). The nomogram incorporated age, C-reactive protein (CRP), Wagner grade, lower extremity arterial disease (LEAD), and peripheral neuropathy (PN). The predictive model exhibited robust discriminatory capacity, achieving an area under the curve (AUC) of 0.803 (95% confidence interval (CI) 0.735–0.878) with internal cross-validation stability (AUC = 0.804). Goodness-of-fit was confirmed by the Hosmer–Lemeshow test (χ^2^ = 5.014, *p* = 0.756), with excellent net benefit shown by decision curve analysis. Our findings indicate a high infection rate in DF patients, mainly caused by *Gram-positive cocci*. The nomogram incorporating age, CRP, Wagner grade, LEAD, and PN parameters enables rapid DFIs screening, facilitating timely antibiotic initiation through early infection detection to enhance clinical management.

## Introduction

Diabetic foot infections (DFIs) is a common and severe complication among diabetic patients. Its high disability and amputation rates impose significant physical and psychological burdens on patients and exert heavy pressure on societal medical resources^1^. These infections, almost triggered by diabetic neuropathy and vascular complications. Therefore, researching the distribution characteristics of pathogenic microorganisms in DFIs and developing risk prediction models is great significance for guiding the rational use of antibiotics in clinical practice, formulating personalized treatment plans, preventing amputations, and improving patient outcomes. Microbiologically, a diverse array of pathogens can cause DFIs, among which *Staphylococcus aureus* emerging as a predominant causative organism. However, the microbial spectrum of DFIs exhibits significant heterogeneity, a phenomenon influenced by various factors such as geographical regions, patient demographic characteristics, and local antimicrobial resistance patterns. Recent studies have emphasized the increasing incidence of multidrug-resistant strains, including *Pseudomonas aeruginosa* and *multidrug-resistant Gram-negative bacilli*, posing significant challenges to empirical antibiotic therapy^2^. Understanding the microbial epidemiology of DFIs is essential for guiding antimicrobial stewardship and optimizing treatment outcomes.

In clinical practice, the diagnosis of DFIs requires a multidimensional approach integrating clinical manifestations, biochemical markers, and advanced imaging modalities to precisely evaluate infection progression and tissue involvement. Current evidence suggests that preventive strategies demonstrate superior cost-effectiveness compared to therapeutic interventions in DFIs management^3^. Early identification, timely diagnosis, and precision treatment can significantly improve the clinical prognosis of patients with DFIs^4^. Consequently, the establishment of rapid-response prediction model is importance for patients with DFIs.

## Methods

### Patients

#### Ethics approval

This retrospective study was approved by the Ethics Committee of the Henan University Huaihe Hospital (Ethics Committee Approval Number:2025008). The patient information was automatically de-identified by the medical record system when the data were collected. The implementation of this study will not have any impact on the treatment and prognosis of the patients. The privacy and rights of the patients are protected. According to the Declaration of Helsinki, the need for informed consent was waived by the Ethics Committee of Henan University Huaihe Hospital. All methods in this study were performed in accordance with the relevant guidelines and regulations and the Declaration of Helsinki.

A total of 186 DF patients who were hospitalized for treatment at Henan University Huaihe Hospital from November 2020 to November 2024 were selected. Their clinical data that include wound culture results, and laboratory test data were collected through the electronic medical record of the hospital information system. The inclusion criteria as follows: (1) Met the 1999 WHO Diabetes Diagnostic Criteria; (2) Comply with the 2023 IWGDF/IDSA guidelines on the diagnosis and treatment of diabetes-related foot infections^5^; (3) The diagnosis of DFIs refers to the relevant diagnostic criteria in Practical Anti-infective Therapy^5,6^; (4) The patient’s clinical data is complete. The Exclusion Criteria as follow: Absence of other causes of skin inflammatory response (e.g., trauma, gout, acute Charcot neuroarthropathy, fracture, thrombosis, or venous stasis); Patients with malignancies; Patients receiving immunosuppressants, glucocorticoids, or chemotherapeutic agents for over 6 months; Patients diagnosed with decompensated cirrhosis.

### Study methods

The age, gender, duration of diabetes, duration of DF, and wagner grade^7,8^ of the patients were collected. Laboratory tests that included white blood cell(WBC), hemoglobin (Hb), low-density lipoprotein cholesterol (LDL-C), high-density lipoprotein cholesterol (HDL-C), serum creatinine (Cr), platelets (PLT), uric acid (UA), fibrinogen (FIB), international normalized ratio (INR), glycated hemoglobin (HbA1c), C-reactive protein (CRP), lower extremity arterial disease (LEAD), and peripheral neuropathy (PN). The diagnosis of LEAD and PN is based on the Chinese Guidelines for the Prevention and Treatment of Type 2 Diabetes^8^.

Before the use of antibiotics in patients with DF, the wound along with the surrounding area was meticulously irrigated with sterile 0.9% sodium chloride solution (normal saline) and 3% hydrogen peroxide, removal of necrotic debris. Specimens were obtained from multiple deep tissue sites and sent for rapid examination. Pathogen culture was performed in accordance with the National Clinical Laboratory Procedures Manual^9^. Suspect colonies were selected, and the test results were interpreted according to the standards and guidelines of the Clinical and Laboratory Standards Institute (CLSI)^10^. In predictive modeling studies, the number of outcome events often determines the effective sample size. Empirical evidence suggests that a robust sample should have at least 10 outcome events per variable (EPV) in multivariate regression analysis, or more precisely, per estimated parameter. Our sample size and the number of events exceeded the threshold determined by the EPV approach for sample size determination. Therefore, we anticipate that our study will provide robust and reliable estimates.

### Statistical analysis

Analysis was performed using R 4.2.1 (http://www.Rproject.org; The R Foundation, Vienna, Austria) and the Free Statistics software (version 2.0; Beijing FreeClinical Medical Technology Co., Ltd, Beijing, China). All statistical tests were 2-tailed, with a significance level of α = 0.05. Continuous data are expressed as mean ± standard deviation (${\overline{\text{x}}}$ ± SD), and comparisons were made using the t-test. Categorical data are presented as proportions or rates (%) and compared using the χ^2^ test. The analysis of influencing factors was performed using a multivariate logistic regression model. Receiver operating characteristic (ROC) curves were plotted to evaluate the predictive performance of individual indicators and the risk prediction model for diabetic foot infections.

We used the least absolute shrinkage and selection operator (LASSO) regression to identify predictive variables for diabetic foot infection. Which were then used to construct a nomogram. The ROC and area under the curve (AUC) were used to evaluate the discriminative ability of the nomogram, and the Hosmer–Lemeshow test was used to evaluate the calibration of the nomogram. The bootstrap method was used for internal validation, Calibration AUC values were calculated, and calibration curves were plotted to assess the predictive ability of the nomogram. Decision curve analysis was used to assess net clinical benefits.

## Results

### General characteristics

**Figure1:**
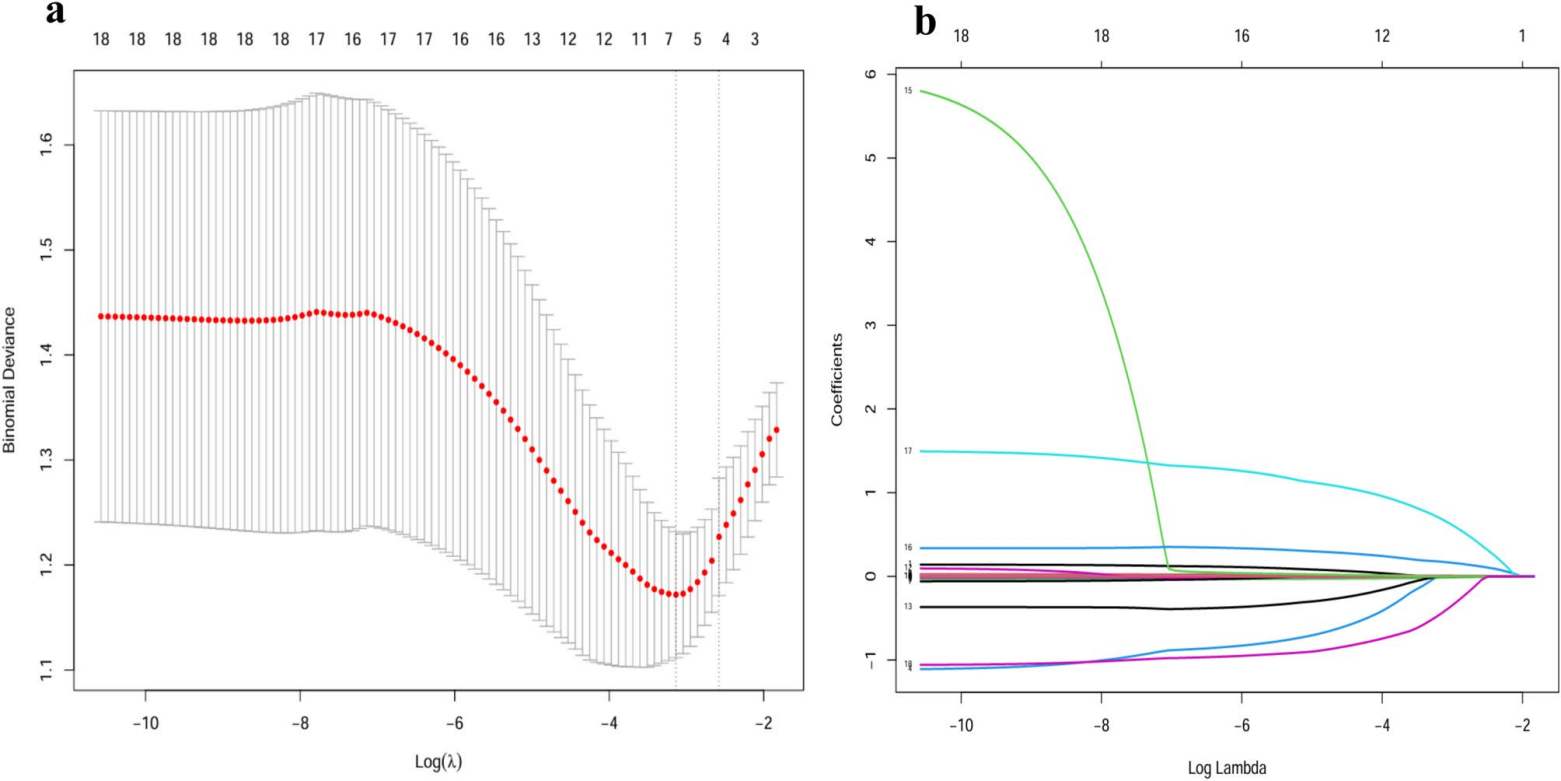
Feature selection using LASSO binary logistic regression model. (**a**) Log (lambda) value of 18 features in the LASSO model. A coefficient profile plot was produced against a log (lambda) sequence. (**b**) Parameter selection in the LASSO model uses five-fold cross-validation through minimum criterion. Partial likelihood deviation (binomial deviation) curves and logarithmic (lambda) curves are plotted. Minimum standard and1-SEof the minimum standard are used to draw a vertical dashed line at the optimal value. Optimal lambda produces 5 nonzero coefficients. LASSO, least absolute shrinkage and selection operator.

A total of 186 patients of DF were reviewed, 50 patients were excluded owing to incomplete data. Finally, 136 patients were included in the study, including 50 non-infection patients (Patients in whom no pathogenic bacteria were detected), and 86 infection patients (Patients in whom pathogenic bacteria were detected). In infection group, the wound culture results showed (n = 47,54.65%) patients infected by *Gram-positive cocci*, including *Staphylococcus aureus* infection (n = 30,34.88%), *Staphylococcus epidermidis* infection(n = 10,11.63%), *methicillin-resistant Staphylococcus aureus (MRSA)* infection (n = 3,3.49%),*Staphylococcus haemolyticus* infection(n = 2,2.33%),*Streptococcus agalactiae* infection(n = 2,2.33%); 37 (43.02%) patients infected by *Gram-negative bacilli* infection, including *Escherichia coli*(n = 18,20.93%), *Pseudomonas aeruginosa* infection (n = 7,8.14%), *Klebsiella pneumoniae* infection (n = 6,6.98%), *Proteus mirabilis* infection (n = 4,4.65%), *Enterobacter cloacae* infection (n = 2,2.33%). *Candida tropicalis* infection (n = 2,2.33%) (Table 1). Of the 18 characteristics (i.e., age, gender, course of diabetes, Course of DF, WBC, CRP, Hb, PLT, Cr, UA, HDL-C, LDL-C, FIB, INR, HbA1c, wagner grade, LEAD, PN) were collected (Table 2). Eight characteristics were selected on the basis of nonzero coefficients calculated by LASSO logistic regression analysis (Fig. 1a,b), The selected features included age, CRP, LEAD, PN, and Wagner grade. These features were then included in the multivariate logistic regression analysis.

**Table 1:** General distribution of infectious bacteria.

Pathogenic bacterial species	Number of cases	Multidrug-resistant bacterial	Proportion (%)
*Gram-positive cocci*	47		54.65
*Staphylococcus aureus*	33	3	34.88
*Staphylococcus epidermidis*	10		11.63
*Staphylococcus haemolyticus*	2		2.33
*Streptococcus agalactiae*	2		2.33
*Gram negative bacilli*	37		43.02
*E. coli*	18	4	20.93
*Pseudomonas aeruginosa*	7	3	8.14
*Klebsiella pneumoniae*	6	2	6.98
*Enterobacter cloacae*	2		2.33
*Proteus mirabilis*	4		4.65
*Fungus*	2		2.33
*Candida tropicalis*	2		2.33

**Table 2:** Characteristics of 136 patients with diabetic foot.

Variables	(n = 136)	Non-infection(n = 50)	Infection(n = 86)	*p*
HbA1c, %	9.6 ± 2.5	9.5 ± 2.7	9.7 ± 2.4	0.664
Age, year	67.2 ± 10.7	70.1 ± 9.6	65.5 ± 10.9	0.015
Gender				0.757
Female	43 (31.6)	15 (30)	28 (32.6)	
Male	93 (68.4)	35 (70)	58 (67.4)	
WBC, *10^9^/L	10.4 ± 5.3	8.7 ± 3.6	11.4 ± 5.8	0.003
Hb, g/L	117.1 ± 23.7	118.7 ± 26.3	116.1 ± 22.1	0.545
PLT, *10^9^/L	271.6 ± 108.2	250.2 ± 99.1	284.1 ± 111.8	0.078
Cr, µmol/L	73.6 ± 40.3	74.7 ± 31.1	72.9 ± 45.0	0.805
UA, µmol/L	283.0 ± 128.6	296.6 ± 126.6	275.1 ± 129.8	0.349
HDL-C, mmol/L	0.9 ± 0.3	0.9 ± 0.3	0.9 ± 0.3	0.101
LDL-C, mmol/L	2.3 ± 0.9	2.5 ± 0.9	2.2 ± 0.9	0.056
FIB, mg/dl	528.7 ± 164.6	475.4 ± 161.3	559.7 ± 159.3	0.004
INR	1.0 (1.0,1.1)	1.0 (1.0,1.0)	1.0(1.0,1.1)	< 0.001
Wagner grade				0.012
1	8 (5.9)	6 (12)	2 (2.3)	
2	23 (16.9)	13 (26)	10 (11.6)	
3	30 (22.1)	10 (20)	20 (23.3)	
4	41 (30.1)	14 (28)	27 (31.4)	
5	34 (25.0)	7 (14)	27 (31.4)	
PN				0.002
No	51 (37.5)	27 (54)	24 (27.9)	
Yes	85 (62.5)	23 (46)	62 (72.1)	
LEAD				0.02
No	21 (15.4)	3 (6)	18 (20.9)	
Yes	115 (84.6)	47 (94)	68 (79.1)	
Course of Diabetes,year	11.5 (8.0, 20.0)	11.0 (8.0, 20.0)	11.5 (8.5, 20.0)	0.96
Course of DF, month	1.0 (0.3, 3.0)	1.0 (0.3, 2.0)	1.0 (0.4, 3.8)	0.581
CRP, mg/L	27.6 (8.0, 95.9)	15.6 (4.8, 44.8)	52.2 (11.6, 130.7)	< 0.001

Data are presented as the median (interquartile range) or n (%). HbA1c, glycated hemoglobin; WBC, white blood cell; Hb, hemoglobin; PLT, platelet; Cr, creatinine; UA, uric acid; HDL, High density lipoprotein; LDL, Low density lipoprotein; FIB, fibrinogen; INR, International standardized ratio; PN, peripheral neuropathy; LEAD, lower extremity arterial disease; CRP, C-reactive protein.

### Risk prediction nomogram development

**Fig. 2:**
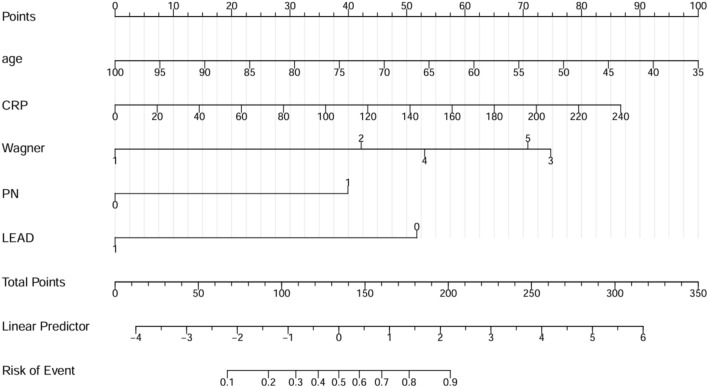
Nomogram for predicting the risk of diabetic foot infection. The first line represents the scoring scale. Corresponding scores for each predictor factor are shown in lines 2–6. The score for each predictor is determined by referencing the first line. The total score for the risk evaluation is the sum of each predictor score. To determine the likelihood of diabetic foot infection, the score point is located on the total point line (line 7). Then, the user descends vertically to the risk of complication (line 9).

Multivariate logistic regression analysis identified age, CRP, LEAD, PN, and Wagner grade as independent predictors of DFIs. These independent predictors were thus incorporated to develop a predictive nomogram (Fig. 2), with a higher score indicating a higher risk of infection. The specific calculation formula for predicting the risk of infection with DF by nomogram was Logit(P) = 2.342–0.051*age + 0.012*CRP + 1.386*(Wagner grade = 2) + 2.454*(Wagner grade = 3) + 1.744*(Wagner grade = 4) + 2.325(Wagner grade = 5) + 1.312* (PN = Yes)-1,699* (LEAD = Yes).

### Performance and validation of the nomogram

**Fig. 3:**
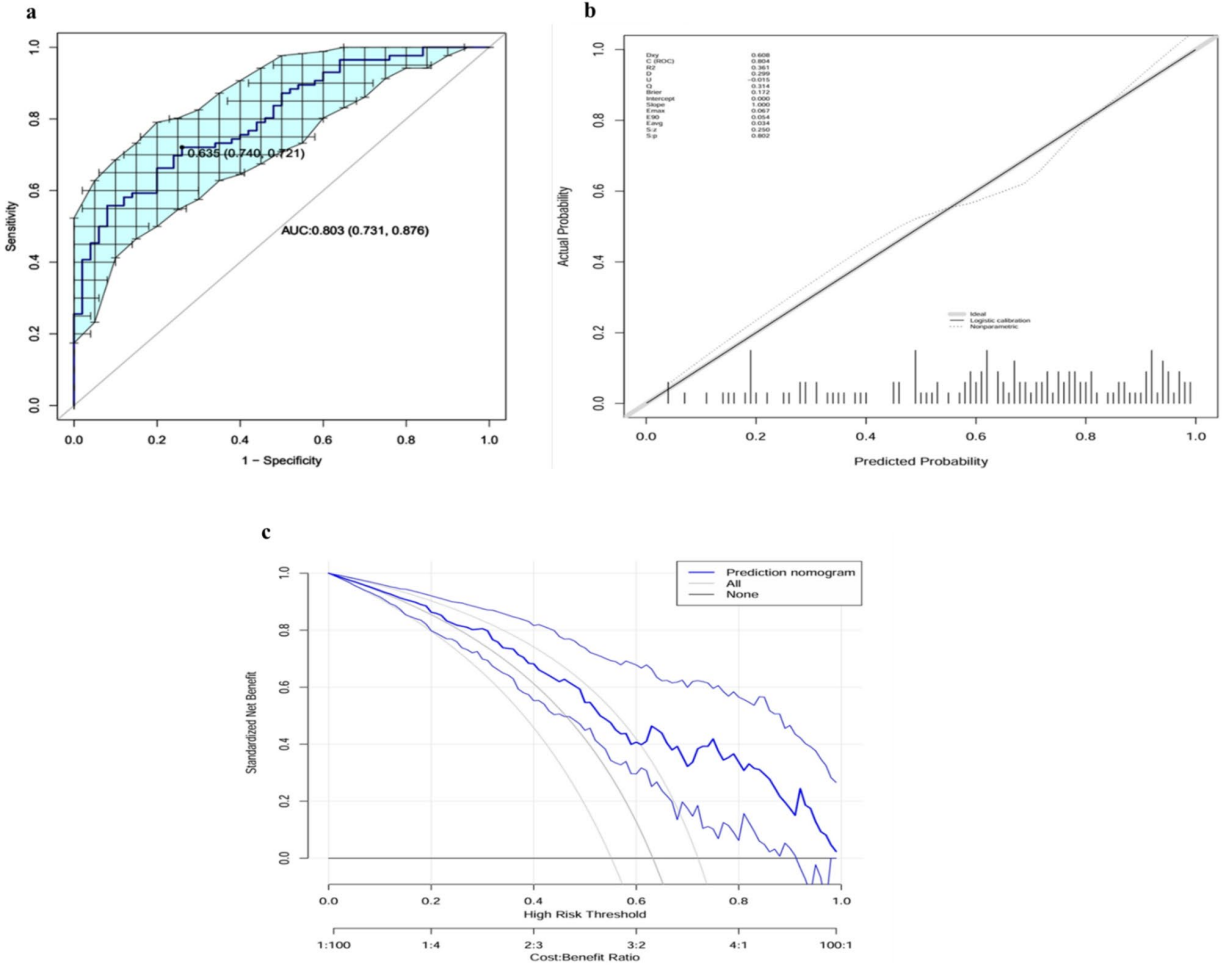
(**a**) ROC curve of the nomogram. The brackets next to the area under the ROC curve (AUC) represent the 95% confidence interval. (**b**) Calibration curve of the nomogram. The apparent curve represents the relationship between predicted and actual probabilities of clinically significant complications. The bias-corrected curve is plotted by bootstrapping using 1000 resamples. The ideal curve is the 45° line, which indicates perfect prediction. (**c**) Decision curve analysis of the nomogram. Blue solid lines represent the nomogram, x axis, cutoff probability, and y axis, net benefit. AUC, area under the curve; ROC, receiver operating characteristic.

The receiver operating characteristic curve showed that the AUC of the nomogram was 0.803 (95% confidence interval (CI) 0.731–0.876) (Fig. 3a). When cutoff value was 0.63, the predictive model’s sensitivity was 0.74, specificity was 0.72, and accuracy was 0.69. Internal validation using the bootstraps method generated an AUC of 0.804(95%CI 0.735–0.878), the calibration curve showed high consistency between the predicted and actual findings (Fig. 3b). In addition, the Hosmer-Leme show test showed a good fit (χ^2^ = 5.014, *p* = 0.756). Decision curve analysis assessment of the clinical utility of the nomogram showed that for all probabilities, applying the nomogram added a more significant net benefit compared with the treat-all or treat-none strategies (Fig. 3c).

## Discussion

DFIs, the severest diabetic complication and a primary cause of disability and amputation, poses significant challenges^11,12^. To facilitate early identification, this study analyzed local DFIs pathogen distribution and developed a DFIs risk prediction model. This model aims to guide antibiotic use, support personalized treatment, prevent amputations, and enhance patient prognosis in clinical settings.

In this study, 63.23% (86/136) of DF patients had infections. *Gram-positive cocci* were the main pathogens (54.65%), with *Staphylococcus aureus* being the most frequent (34.88%). *Gram-negative bacteria* infections accounted for 43.02%, mostly *Escherichia coli* (20.93%). *Pseudomonas aeruginosa* and *Klebsiella pneumoniae* infections made up 8.14% and 6.98% of cases, respectively. Notably, 10.47% of infections were from *multidrug-resistant Gram-negative bacteria*, and 3.49% were *MRSA* infections, highlighting the ongoing threat of *multidrug-resistant bacteria* (*MDRB*) in DFIs^13^. The distribution of pathogens causing DFIs is consistent with previous research findings^14,15^. However, there are minor discrepancies in the proportions, which are presumably attributable to regional disparities, demographic differences, and variations in antibiotic usage. In our region, *Staphylococcus aureus* emerges as the predominant pathogen. Consequently, it is advisable to commence empirical penicillin—based therapy while awaiting the results of wound cultures. It is noteworthy that *multidrug—resistant Gram—negative bacterial* infections pose a significant challenge. When selecting antibiotics, a targeted approach should be adopted, along with ensuring appropriate dosage and sufficient treatment duration^16–19^.

Based on age, CRP, LEAD, PN, and Wagner grade. We developed a nomogram for assessing the risk of DFIs. The model’s performance was validated through three aspects: internal cross—validation, decision—curve analysis, and the Hosmer—Lemeshow test, all of which indicated good performance. Admittedly, The cohort contained 86 event-positive cases (63%, N = 136), creating substantial asymmetry in outcome distribution. In such imbalanced settings, over-reliance on sensitivity alone becomes inadequate for assessing discriminative capacity. Our multiparametric evaluation framework incorporated: PPV is 92%, the AUC is 0.803 (95% CI 0.731–0.876), Hosmer Lemeshow test (χ^2^ = 5.01, *p* = 0.756). These metrics collectively confirm satisfactory calibration-discrimination balance per TRIPOD guidelines. The inverse sensitivity–specificity relationship necessitated clinical compromise, When cutoff as 0.63, Sensitivity was 74% and specificity was 72%; achieving optimal net benefit in decision curve analysis (DCA) between 0.2 and 0.8 risk probabilities. Moreover, compared to the infection assessment methods in the guidelines^5^, the variables incorporated in the model are more objective and comprehensive.

## Limitations

Although significant progress has been made in the analysis of pathogen distribution characteristics and the development of risk prediction models in this study, there are still some limitations. Firstly, this study is a single center study with a relatively small sample size, which may limit the generalizability of the results. Future research should consider multi center, large sample study designs to improve the extrapolation of results. Secondly, although the risk prediction model we developed performed well in internal validation, its performance in external datasets still needs further validation. Future research should consider external validation in different regions and populations to evaluate the model’s generalization ability. In addition, this study did not conduct a detailed analysis of the drug resistance of pathogens. Future research should combine drug resistance data to further optimize risk prediction models. Finally, this study did not analyze the long-term follow-up data of patients. Future research should consider including long-term follow-up data to evaluate the predictive ability of the model for long-term prognosis of patients.

## Conclusion

This study developed a risk prediction model based on clinical characteristics by analyzing the distribution characteristics of pathogenic bacteria of diabetes foot infection. This model demonstrates good discriminative ability and clinical utility in predicting the risk of DFIs, providing important references for the rational use of antibiotics and the development of personalized treatment plans in clinical practice. In addition, this study emphasizes the importance of multidrug-resistant strains in DFIs, suggesting that clinical doctors should pay more attention to drug resistance issues in the treatment process to improve treatment efficacy and patient prognosis.

## Data Availability

The data that support the findings of this study are not openly available due to reasons of sensitivity and are available from the corresponding author upon reasonable request.
